# A methodological approach for collecting simultaneous measures of muscle, aponeurosis, and tendon behaviour during dynamic contractions

**DOI:** 10.1242/bio.060383

**Published:** 2024-05-23

**Authors:** Stephanie A. Ross, Christine Waters-Banker, Andrew Sawatsky, Timothy R. Leonard, Walter Herzog

**Affiliations:** Human Performance Laboratory, Faculty of Kinesiology, University of Calgary, Calgary, AB T2N 1N4, Canada

**Keywords:** Biomechanics, Skeletal muscle, Tendon, Aponeurosis, Sonomicrometry, Dynamic contractions

## Abstract

Skeletal muscles and the tendons that attach them to bone are structurally complex and deform non-uniformly during contraction. While these tissue deformations dictate force production during movement, our understanding of this behaviour is limited due to challenges in obtaining complete measures of the constituent structures. To address these challenges, we present an approach for simultaneously measuring muscle, fascicle, aponeurosis, and tendon behaviour using sonomicrometry. To evaluate this methodology, we conducted isometric and dynamic contractions in *in situ* rabbit medial gastrocnemius. We found comparable patterns of strain in the muscle belly, fascicle, aponeurosis, and tendon during the isometric trials to those published in the literature. For the dynamic contractions, we found that our measures using this method were consistent across all animals and aligned well with our theoretical understanding of muscle-tendon unit behaviour. Thus, this method provides a means to fully capture the complex behaviour of muscle-tendon units across contraction types.

## Teaser

**Summary:** This paper presents a new method to simultaneously measure muscle, aponeurosis, and tendon properties during contractions that are relevant to locomotion.

## INTRODUCTION

Muscle-tendon units (MTUs), which are composed of skeletal muscle, aponeurosis, and tendon, have a complex three-dimensional (3D) structure that deforms non-uniformly during contraction. Tendon attaches muscle to bone on both distal and proximal ends, and aponeurosis, a thin sheet of viscoelastic material that is a continuation of the tendon, covers the superficial and deep surfaces of many pennate muscles. The muscle itself is composed of bundles of fibres called fascicles that in unipennate muscle, like the much-studied medial gastrocnemius, run from the deep to the superficial aponeurosis at an angle relative to the muscle force-generating axis. For an isometric contraction in which the joint angles and muscle-tendon unit length are held constant, the muscle belly shortens when activated and the fascicles shorten and rotate (Herbert and Gandevia, 1995; Huijing and Woittiez, 1984; Maganaris and Baltzopoulos, 1982; Muhl, 1982; Narici et al., 1996). The active muscle force transmits to the tendon, causing it to stretch to a longer length (Cui et al., 2009; Griffiths, 1991; Monti et al., 2003; Scott and Loeb, 1995). Although aponeurosis behaviour is poorly described, it appears to lengthen and behave similar to a tendon during isometric conditions (Ettema and Huijing, 1989; Huijing and Ettema, 1988; Lieber et al., 2000; Monti et al., 2003; Scott and Loeb, 1995; Zuurbier et al., 1994). While this pattern of deformation is typical of isometric contractions, how and when the constituent structures deform can vary widely during locomotor activities depending on the material and structural properties of the particular MTU, the pattern of muscle activation, and the applied external loads.

Our understanding of MTU behaviour during dynamic conditions that are relevant to locomotion is limited, as most studies have measured only a select number of structures at once during isometric contractions, in part due to limitations of current measurement techniques. In humans, ultrasound is typically used to measure dynamic MTU behaviour *in vivo* (Dick and Hug, 2023). While ultrasound is affordable and non-invasive, a single probe only provides a two-dimensional (2D) image of muscle with a field-of-view that is often smaller than necessary to image an entire fascicle, let alone an entire MTU. Measuring aponeuroses using ultrasound is also challenging given the lack of distinct landmarks that can be tracked across frames. Magnetic resonance imaging (MRI) is increasingly being used to visualise the 3D structure of *in vivo* human muscle during isometric conditions. However, the time resolution of MRI is too low to provide accurate measures of dynamic contractions, and the contrast between muscle and aponeurosis is often insufficient to differentiate between the two tissues (Wakeling et al., 2020). Thus, approaches used to measure MTU behaviour in humans are not able to fully capture the 3D deformations of all constituent structures during dynamic contractions.

Animal studies have provided more detailed descriptions of MTU behaviour during dynamic contractions, as more invasive and direct measurement approaches can be used in these preparations. Sonomicrometry is one such approach that involves measuring the distance between pairs of piezoelectric crystals implanted in the tissue via the transit time of sound waves from one crystal to the other (Griffiths, 1987). Most studies using sonomicrometry only use a maximum of two crystal pairs (four total) within a given muscle; for example, crystals are often placed at the ends of a representative fascicle to examine the uncoupling of fascicle and muscle length change (Azizi and Roberts, 2014; Azizi et al., 2008; Holt et al., 2016). Of the studies that have utilised a greater number of crystals to measure more structures simultaneously, most have only done so during isometric conditions in which the deformations of the tissue are likely smaller and occur at a slower rate than those that occur during dynamic contractions. Within the last decade, other approaches have been developed to measure MTU behaviour *in vivo*, including fluoromicrometry (Camp et al., 2016) and magnetomicrometry (Taylor et al., 2021, 2022). While fluoromicrometry allows numerous radio-opaque beads to be tracked using biplanar x-ray to fully capture 3D tissue deformations, this tracking is labour and time intensive, which limits how many structures can feasibly be measured at once (Roberts and Dick, 2023). Despite the promise of the newer magnetomicrometry, which tracks implanted magnetic beads in an array of magnetic field sensors, this approach has not been extensively tested and validated. Therefore, as with approaches used in human studies, current methods of measuring MTU behaviour during dynamic conditions in animals are limited in their ability to capture all component structures.

In this study we examined the feasibility and utility of expanding on current sonomicrometry approaches to measure a greater number of MTU structures during dynamic contractions by increasing the number of implanted crystals to better capture the 3D behaviour of the whole system. We tested this approach on *in situ* rabbit medial gastrocnemius muscle during dynamic shortening and lengthening contractions, as well as during isometric contractions to relate our measurements to those of previous studies.

## RESULTS

**Fig. 1.**
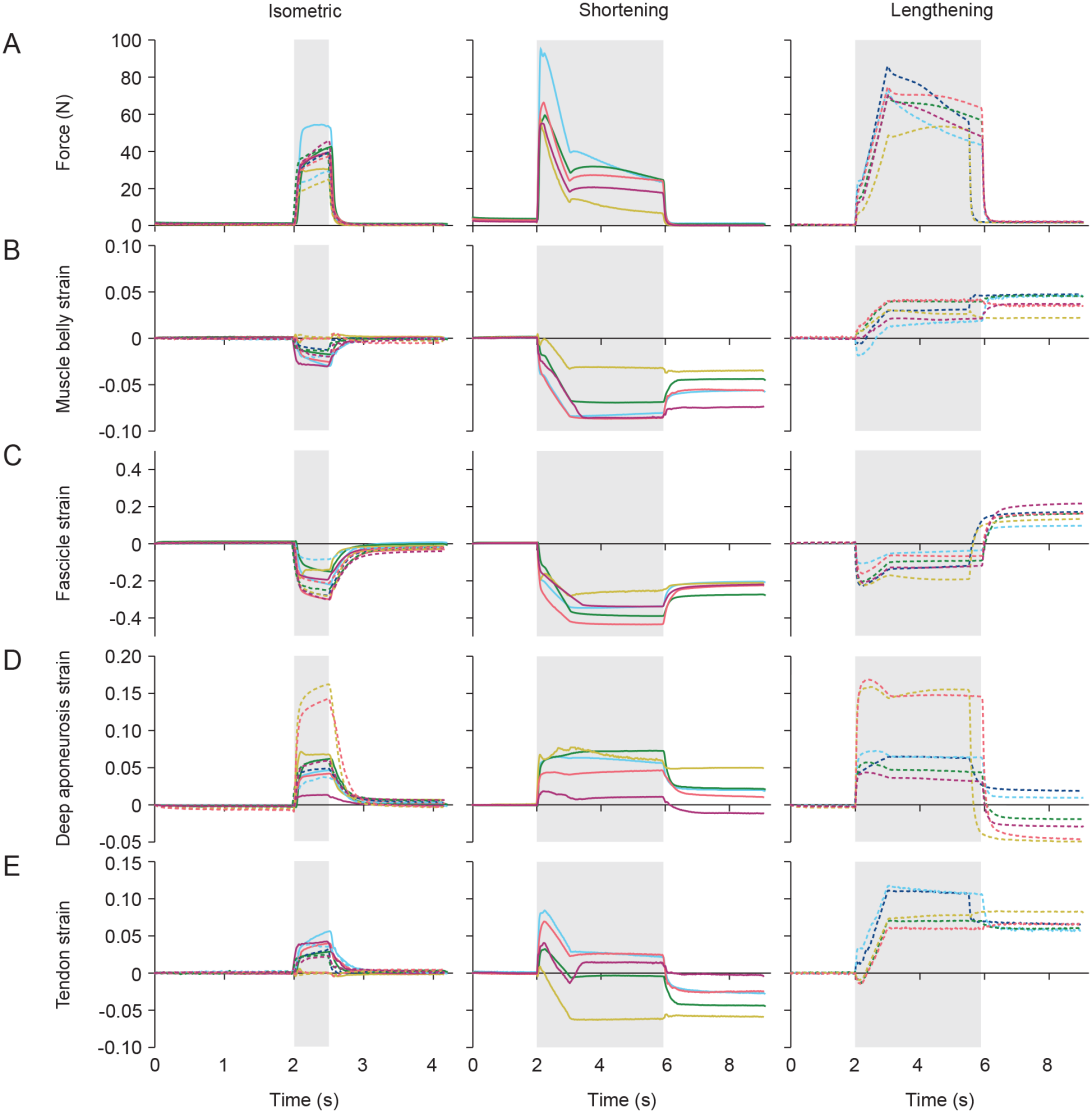
**Force (A), muscle belly strain (B), fascicle strain (C), deep aponeurosis strain (D), and tendon strain (E) over time for isometric (left; *n*=11), shortening (middle; *n*=5), and lengthening (right; *n*=6) contractions.** Strain values are unitless and given by the change in length between crystal pairs relative to the reference length. All tissue length changes were measured from the sonomicrometry except for the tendon, which was calculated from the motor and muscle belly displacements. The region shaded in grey indicates the time period in which the nerve was stimulated. Each colour-dash combination represents data from a different animal.

For isometric contractions with fixed MTU length, we found that force increased with stimulation of the nerve and subsequent activation of the muscle (Fig. 1A, left). This increase in force corresponded with a decrease in muscle belly and fascicle length and an increase in deep aponeurosis and tendon length across the 11 rabbits (Fig. 1B-E, left), except for three in which the belly and tendon lengths remained largely isometric (Fig. 1B,E, left). We found evidence of larger mean peak strain in the deep aponeurosis of 6.8±1.4% compared to the tendon of 2.7±0.5% for the active isometric contractions (*P*=0.047, t=2.26, d.f.=10). We also found that the mean peak strain of the representative fascicle was far larger than that of the muscle belly at −22.4±2.1% compared to 1.7±0.5% (*P*<0.001, t=9.74, d.f.=10). Once the nerve stimulation was removed and force decreased, the muscle belly, fascicle, deep aponeurosis, and tendon returned to close to their initial lengths.

For the shortening contractions, muscle belly and fascicle strain decreased during the active shortening phase and remained at shorter strains relative to initial during the active and isometric hold periods following shortening (Fig. 1B,C, middle). Both deep aponeurosis and tendon strain increased as the muscle force increased with activation (Fig. 1D,E, middle). Aponeurosis strain stayed relatively constant during the active shortening phase and did not shorten again until the muscle became passive (Fig. 1D, middle). In contrast, tendon strain followed a similar trajectory to muscle force in that its strain decreased as force decreased during shortening and then stayed fairly constant during the active isometric hold following shortening (Fig. 1E, middle).

For the lengthening contractions, there was an uncoupling of belly and fascicle strain during the active lengthening phase such that muscle belly strain increased, and fascicle strain decreased (Fig. 1B,C, right). Upon muscle deactivation at the longer final length, both belly and fascicle strain remained higher relative to their initial length before active lengthening. Both deep aponeurosis and tendon strain increased during active lengthening when the force and length of the MTU increased (Fig. 1E,D, right). When the muscle was deactivated at the longer final length, aponeurosis strain returned to close to its resting values when the MTU was at its short initial length compared to the tendon which remained stretched relative to its initial length.

## DISCUSSION

In this study we aimed to evaluate the feasibility and utility of measuring the strain of numerous MTU structures simultaneously using sonomicrometry, including the muscle belly, representative fascicle, deep aponeurosis, and distal tendon during dynamic contractions. We additionally conducted isometric MTU contractions to evaluate our measures against those of previous studies which primarily focused on contractions with fixed MTU length. Across all contraction types, we found consistent patterns of strain across all structures to what has been reported previously in the literature, as well as fairly consistent patterns across the animals in this study (Fig. 1). For isometric contractions with constant MTU length, both the fascicle and muscle belly shortened while the deep aponeurosis and tendon lengthened, as expected (Scott and Loeb, 1995). The mean peak fascicle strain was substantially larger than that of the muscle belly (−22.4% versus −1.7%), which likely occurred due to the increases in fascicle angle and decreases in the belly thickness that have been reported in the medial gastrocnemius (MG) (Randhawa and Wakeling, 2015; Randhawa et al., 2013).

We found a mean maximum aponeurosis strain of 6.8%, which was greater than the 2.7% strain in the tendon for the isometric contractions. This difference in maximum strain between aponeurosis and tendon is consistent with findings of previous studies that report values for both tissues in the same preparation (Ettema and Huijing, 1989; Lieber et al., 1991; Maganaris and Paul, 2000a,b; Monti et al., 2003; Muramatsu et al., 2001). However, our values were on the lower end of more widely reported maximum strains during isometric contractions at or near muscle optimal length ranging from less than 1% (van Donkelaar et al., 1999) to 14% (Zuurbier et al., 1994) for aponeurosis and from 2% (Lieber et al., 1991) to 9% (Adam et al., 2023; Waugh et al., 2012) for tendon. The wide range of reported strains likely reflects differences in muscles and species, measurement techniques, and methods of calculating and reporting strain. Engineering strain of an elastic tissue is given relative to its slack length, which is often difficult to determine, particularly during *in vivo* conditions and for intact aponeurosis where the tissue may never be truly unloaded. As a result, some studies report strain relative to the tendon length at muscle optimal length (e.g. van Donkelaar et al., 1999) or to an extrapolated slack length (e.g. Zuurbier et al., 1994), which may underestimate or overestimate maximum strain, respectively. In our study, we defined the reference length as the length where 2 *N* of passive force was reached, which would correspond to approximately slack length of the tendon or slightly longer. This may explain our maximum strain values that are on the lower end of those that have been reported in previous studies.

For the shortening contractions, tendon strains closely followed changes in force in that the tendon stretched to longer lengths when the force was high and recoiled when force decreased, consistent with the ubiquitous notion that tendon acts as a (nearly) elastic element in series with muscle (Hill, 1938, 1950). In contrast to the tendon, the aponeurosis stretched with muscle activation, then stayed at a constant length during the active shortening and active isometric phases. Because aponeurosis overlays the surface of muscle, it likely experiences more complex and multidirectional loading than tendon (Arellano et al., 2016; Azizi and Roberts, 2009; Epstein et al., 2006; Scott and Loeb, 1995; van Donkelaar et al., 1999), which may contribute to differences in behaviour between the structures. For the shortening and lengthening contractions, we saw that resting tendon strain at the end of the trial was shorter and longer, respectively, than the initial tendon strain (Fig. 1E). Although the muscle would be at a different position on its force-length curve, the differences between initial and final passive forces were small (Fig. 1A). But given that tendon undergoes large strains with small increases in force at short lengths and becomes stiffer at long lengths (Pollock and Shadwick, 1994), the tendon was likely operating in its more compliant region, resulting in fairly large differences in initial and final strain despite small differences in force. Overall, the results from our dynamic contractions align well with our theoretical understanding of MTU behaviour.

While we attempted to measure tendon strain throughout the contractions using sonomicrometry, the quality of the signal declined as the tendon started to move. As a result, we measured the tendon strain using displacements of the motor and muscle belly relative to initial tendon length from the sonomicrometry. In this case, it was likely that tendon displacement resulted in movement of the crystals relative to the tendon that impeded the signal, which may have been addressed by placing the crystals in a holder (e.g. Griffiths, 1987). To minimise this movement artifact in the tendon signal, it is tempting to consider implanting the crystals directly into the tendon tissue as is done with muscle. However, previous authors have suggested that the substantial changes in the stiffness of tendon across its operating range could alter ultrasound transit times between crystals independent of the distance between them (Griffiths, 1991; Marsh, 2016), so tendon measures from implanted crystals may not be accurate even if they appear high quality. While alternative length gauge sensors, such as those composed of silastic tubing filled with mercury (Prochazka et al., 1974; Whitney, 1953) or saline (Hoffer, 1990; Loeb et al., 1980), have been used to measure *in vivo* muscle lengths, these sensors can suffer from drift due to temperature sensitivity and inward diffusion of interstitial fluid (Weytjens et al., 1992). *In vivo* tendon lengths have been measured using fluoromicrometry in flying bats (Konow et al., 2015) and running rats (Konow et al., 2020); however, the small capture volume of this method limits its use to smaller animals. Thus, the search for an ideal method of measuring tendon lengths across preparation types and animal sizes remains.

Whilst we present 2D measures of MTU behaviour in this paper to relate to the results of previous studies, our approach could easily be extended to improve our understanding of 3D muscle behaviour during contraction. In the last 20 years, the development and use of 3D continuum models has highlighted the importance of considering the effect of more complex 3D tissue deformations on the mechanical output of muscle. For example, these models have been used to show the interplay between factors such as regional activation (Rahemi et al., 2014), transverse compression (Ryan et al., 2020), tissue inertia (Ross et al., 2021), and complex multipennate architecture (Knaus et al., 2022) with muscle 3D deformations and force and work output. Our approach highlights the usefulness of sonomicrometry in providing simultaneous measures of multiple dimensions, and further studies could aim to modify or extend on our presented crystal arrangement to allow for more detailed descriptions of 3D muscle deformations. This would not only provide further insight into the determinants of muscle performance but would also provide data that could be used to validate 3D muscle models.

Measuring MTU behaviour and its constituent structures during dynamic contractions is a substantial challenge in the field of biomechanics. Many current approaches have limitations that prevent simultaneous measures of deformations of the entire system, such as measures being restricted to a 2D plane, small capture volumes, and labour and time intensive data analysis. To address these limitations, we evaluated the feasibility and utility of measuring numerous MTU structures simultaneously using sonomicrometry, including the muscle belly, representative fascicle, aponeurosis, and tendon. Although sonomicrometry is not without its own limitations, such as tethered cables and challenges in measuring tendon behaviour, we found that this approach can be used to capture more complex MTU behaviour during dynamic contractions, particularly of the aponeurosis, which is poorly understood.

## MATERIALS AND METHODS

### Animals and surgical preparation

We collected measures of muscle, fascicle, aponeurosis, and tendon behaviour from the right MG of skeletally mature New Zealand white rabbits (*n*=11, mean mass±s.d.=5.1±0.3 kg, Riemens Fur Ranches, St. Agatha, ON, Canada). We chose rabbits for this study as they are a commonly used species, and their muscles are sufficiently large to accommodate the implanted sensors. All surgical and experimental procedures were approved by the Animal Care Committee of the University of Calgary (protocol: AC11-0035).

Rabbits were sedated with 0.18 mL (10 mg/ml) acepromazine (acepro-25; Vétoquinol Inc, Lavaltrie, QC, Canada) and deeply anaesthetised with 1.5% isoflurane in 1 l/min O_2_ administered with a mask. To externally stimulate the muscle, we placed a custom-made bipolar nerve cuff electrode around the tibial nerve which we accessed through the lateral aspect of the thigh. We then opened the posterior aspect of the lower leg from the popliteal fossa to the calcaneus and used blunt dissection to expose and separate the MG muscle and its tendon from surrounding tissue. To reduce the impact of unwanted co-contraction of surrounding muscles, we cut the distal tendons of the lateral gastrocnemius, soleus, and plantaris. We secured rabbits to a stainless-steel stereotaxic frame which fixed the hip and knee angles, cut the calcaneus to leave the tendon intact, and secured the remnant piece of the calcaneus to a muscle motor (MTS; Eden Prairie, MN, USA; natural frequency>10 kHz). While the hip and knee angles were fixed at 120 and 90 degrees, respectively, since we removed and fixed the calcaneus to the muscle motor, the MTU length and behaviour was independent of joint angle. We tented the skin of the lower leg and applied warm saline and ultrasound gel to keep the muscle hydrated and used a heat mat and infrared lamp to maintain the muscle temperature between 30-35°C. Following the experiments, we euthanised the deeply anaesthetised animals with an overdose of intravenously injected pentobarbital sodium (Euthanyl; MTC Pharmaceuticals; Cambridge, ON, Canada).

### Sonomicrometry

**Fig. 2.**
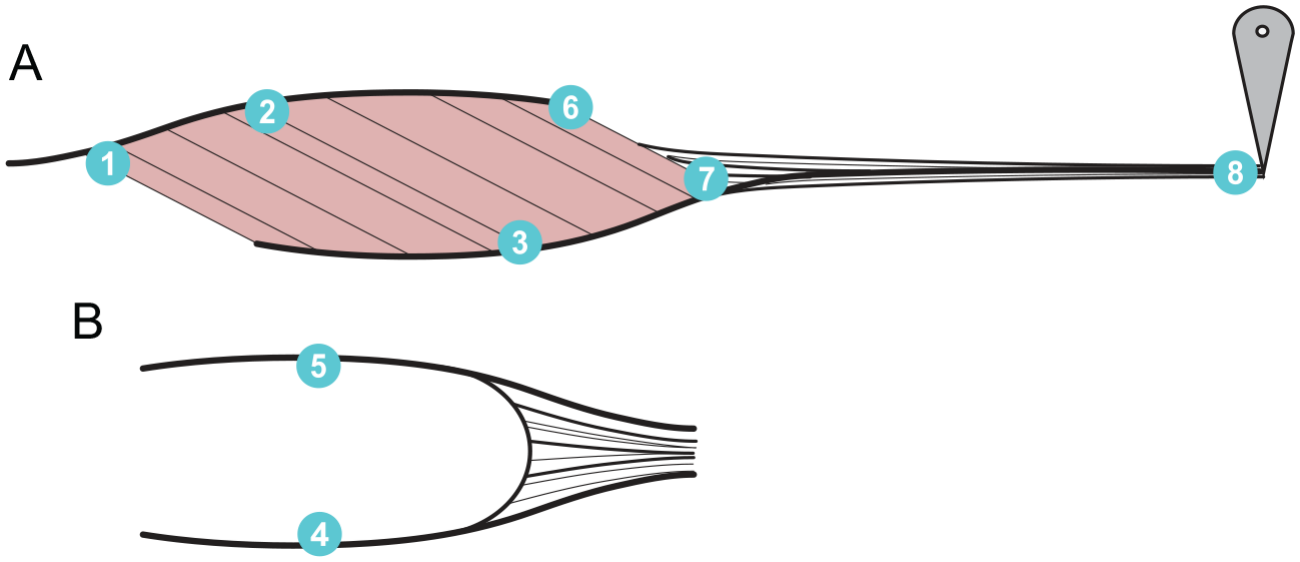
**Placement of sonomicrometry crystals within the muscle-tendon unit.** (A) Side view; (B) superficial view. Lengths relevant to the results presented in this paper are given by the distances between the following crystal pairs: 1 to 7: muscle belly length; 2 to 3: representative fascicle length; 3 to 7: deep aponeurosis length; and 7 to 8: tendon length.

We used sonomicrometry (Sonometrics Corporation, London, ON, Canada) to collect simultaneous measures of the MG muscle, fascicle, and aponeurosis, and tendon behaviour during contraction. The arrangement of the crystals can be seen in Fig. 2. In brief, we implanted and sutured eight piezoelectric crystals into the MG MTU: (1) proximal end of muscle, (2) superficial/proximal end of proximal fascicle, (3) deep/distal end of proximal fascicle, (4) medial side of superficial aponeurosis, (5) lateral side of superficial aponeurosis, (6) distal end of superficial aponeurosis, (7) distal end of muscle, and (8) distal end of tendon. Lengths relevant to the results presented in this paper are given by the distances between the following crystal pairs: 1 to 7: muscle belly length, 2 to 3: representative fascicle length, 3 to 7: deep aponeurosis length, and 7 to 8: tendon length. We sutured crystal (8) to the outer surface of the tendon so that the signal would propagate through the water and ultrasound gel bath rather than through the tendon tissue. The aponeurosis crystals were embedded just deep to the aponeurosis and then sutured to the aponeurosis such that their movement would be dictated by the aponeurosis but their signal would propagate through the muscle tissue. We determined the location of crystals (2) and (3) using muscle surface microstimulation (Griffiths, 1987). To implant crystals (1) to (7), we made a small incision in the tissue, inserted the crystal, then used a single suture to close the incision and prevent movement of the crystal. To further prevent crystal movement relative to the tissue, we made the incisions small enough such that the crystals fit snuggly in the tissue and ensured that there was no tension in the crystal cables that could dislodge them during the trials. Crystals (1), (7), and (8) were 2 mm in diameter and crystals (2) to (6) were 1 mm.

### Testing protocol

The data presented here were collected as part of a larger study in which each muscle was subjected to a range of supramaximal contractions. Therefore, to avoid excessive muscle fatigue and damage due to repetitive contraction, each rabbit underwent isometric and either shortening (*n*=5) or lengthening (*n*=6) contractions. The following description of trials relates only to those that we present results for in this paper.

**Fig. 3.**
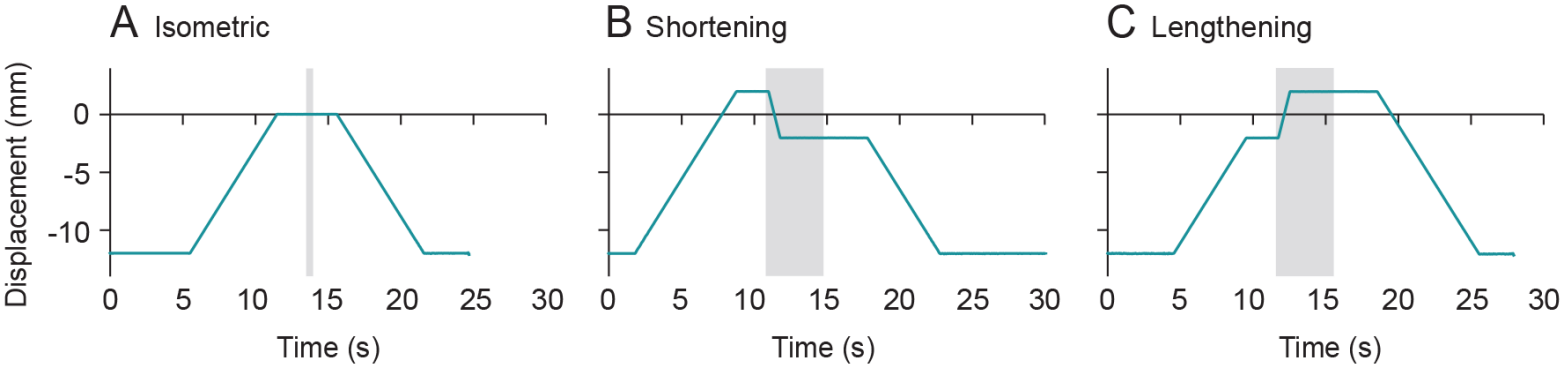
**Traces of motor displacement relative to the reference length over time for the isometric (A), shortening (B) and lengthening contractions (C).** The region shaded in grey indicates the time period in which the nerve was stimulated.

Before starting the contraction trials, we determined the stimulation voltage required to achieve supramaximal tetanus and held the voltage constant at this value for the duration of the experiment. We then conducted isometric contractions (70 Hz stimulation frequency; 0.1 ms pulse duration; Grass S8800, Astro/Med Inc., Longueil, QC, Canada) at a range of fixed MTU lengths relative to a reference length where the passive force was 2 *N*, which we defined as a length of 0. Between all contractions, the muscle was returned and held at a resting length of −12 mm. For shortening contractions, we passively stretched the MG MTU from the resting length to a length of 2 mm, stimulated the muscle for 200 ms at this fixed MTU length, then shortened the muscle with a velocity of 5 mm/s to a final length of −2 mm. Following the shortening period, the MG actively held at the final MTU length for an additional 3 s and then shortened back to the resting length. We used this same protocol for the lengthening contractions, except that the initial and final lengths were interchanged such that the muscle was actively stretched rather than shortened. See Fig. 3 for a visual of the prescribed MTU lengths over time for each contraction type.

Forces and displacements of the muscle motor and stimulation sync pulses were continuously sampled at 500 Hz using WinDaq data acquisition software and DI-400 USB DAQ (Dataq Instruments, Akron, OH, USA). Sonomicrometry was continuously sampled at 440 Hz in SonoLab (Sonometrics Corporation) with a default velocity of sound setting of 1540 m/s for all crystals. To time synchronise the forces, displacements, nerve stimulations, and sonomicrometry data, we used the rising edge of a sync pulse that was recorded simultaneously in WinDaw and SonoLab.

### Data and statistical analysis

Following time synchronisation of the signals and removing artifacts from the sonomicrometry data using SonoCleaner (Chen and Herzog, 2018), forces and displacements from the motor and sonomicrometry data were low-pass filtered using a zero-phase 4th order Butterworth filter with a cut-off frequency of 30 Hz. We calculated time-varying strains from the distances between crystal pairs as the instantaneous change in length relative to the reference length where the passive force was equal to 2 *N*, which approximately corresponded to the muscle and tendon slack length. Because the quality of the sonomicrometry data for the tendon during active contraction was poor, we only used the resting length from these traces and calculated the tendon length change during the contractions as the difference between the motor and muscle belly displacements.

To confirm that our method of measuring the behaviour of MTU components results in similar patterns of strain to those reported in previous studies, we examined differences in peak strain from reference length during the isometric contractions between the tendon and deep aponeurosis and between the muscle belly and representative fascicle. To accomplish this, we assessed differences in sample pairs using paired *t*-tests in R (version 4.3.2; www.R-project.org). All results are reported as means±standard error of the mean.
